# Microparticles as Viral RNA Carriers from Stool for Stable and Sensitive Surveillance

**DOI:** 10.3390/diagnostics13020261

**Published:** 2023-01-10

**Authors:** Emmanuel George Kifaro, Mi Jung Kim, Seungwon Jung, Yoon-ha Jang, Sungyeon Moon, Dong-Hun Lee, Chang-Seon Song, Gerald Misinzo, Sang Kyung Kim

**Affiliations:** 1 Molecular Recognition Research Center, Korea Institute of Science and Technology (KIST), Seoul 02792, Republic of Korea; 2 Department of Veterinary Microbiology, Parasitology, and Biotechnology, Sokoine University of Agriculture (SUA), Morogoro P.O. Box 3019, Tanzania; 3 SACIDS Africa Centre of Excellence for Infectious Diseases, SACIDS Foundation for One Health, Sokoine University of Agriculture (SUA), Morogoro P.O. Box 3297, Tanzania; 4 College of Veterinary Medicine, Konkuk University, Seoul 05029, Republic of Korea; 5 KHU-KIST Department of Converging Science and Technology, Kyung Hee University, Seoul 02447, Republic of Korea

**Keywords:** hydrogel microparticles, viral RNA carrier, qPCR, influenza A virus, stool samples

## Abstract

Since its discovery, polymerase chain reaction (PCR) has emerged as an important technology for the diagnosis and identification of infectious diseases. It is a highly sensitive and reliable nucleic acids (NA) detection tool for various sample types. However, stool, which carries the most abundant micro-organisms and physiological byproducts, remains to be the trickiest clinical specimen for molecular detection of pathogens. Herein, we demonstrate the novel application of hydrogel microparticles as carriers of viral RNA from stool samples without prior RNA purification for real-time polymerase chain reaction (qPCR). In each microparticle of primer-incorporated network (PIN) as a self-sufficient reaction compartment, immobilized reverse transcription (RT) primers capture the viral RNA by hybridization and directly initiate RT of RNA to generate a pool of complementary DNA (PIN-cDNA pool). Through a simple operation with a portable thermostat device, a PIN-cDNA pool for influenza A virus (IAV) was obtained in 20 min. The PIN-cDNA pools can be stored at room temperature, or directly used to deliver cDNA templates for qPCR. The viral cDNA templates were freely released in the subsequent qPCR to allow amplification efficiency of over 91%. The assay displayed good linearity, repeatability, and comparable limit of detection (LoD) with a commercialized viral RNA purification kit. As a proof of concept, this technology carries a huge potential for onsite application to improve human and animal infectious disease surveillance activities using stool samples without the need for a laboratory or centrifuge for sample preparation.

## 1. Introduction

The breakthrough in molecular diagnostics in the field of virology has revolutionized the detection and characterization of viruses [1,2]. The nucleic acids (NA) amplification technologies have become the cornerstone for viral NA analysis. They have been used for the qualitative detection of the presence of viral pathogens in specimens, quantification of the viral particles, and nucleotide sequencing to deduce their genetic diversity [2,3]. Despite the increase in variates of these technologies, real-time PCR (qPCR) is widely used today and is considered the gold standard due to its reliability and high sensitivity [1,4,5,6]. Their analytical efficiency, to a great extent, depends on the quality of NA extracted from biological samples, which is a critical step to facilitate their enzymatic amplification and reliability of test results [3]. Conventional qPCR and reverse transcription qPCR (RT-qPCR) platforms rely on commercially available NA purification kits, which use magnetic beads coated with silica or silica-based membrane filters in a column (e.g., spin-column of the Qiagen kits). Despite the vast advantages of PCR and spin-column extraction kits, their application in resource-limited areas has been limited due to several factors including; the need for highly sophisticated laboratory infrastructure and instruments, a stable cold chain for sample and reagents storage, and the requirement for accessory devices (e.g., centrifuge) [4,7,8]. Apart from spin column-related kits which are solid-phase systems where NA adsorption depends on either silica matrix under chaotropic conditions, ionic exchanges, affinity, or size exclusion mechanisms are currently the preferred NA purification methods for research and diagnostic purposes [9,10]. The other methods include precipitation, liquid–liquid extraction techniques (e.g., phenol–chloroform) which were among the earliest NA purification methods. These platforms are either automated or manually operated to improve the NA yield while removing PCR inhibitory substances for successful NA amplification [11]. In addition to their success, they remain to be either complicated, labor-intensive, or time-consuming. For example, the use of manually operated spin column kits is still challenging, as having numerous sample preparation steps increases the risk of NA degradation, sample loss, and cross-contamination [10]. Higher NA yield and quality can be achieved by using automated extraction platforms, which have demonstrated minimal inter-operator variability and hands-on time [12,13]. The use of these classical platforms comes with increased operational costs, and additional equipment requirements (e.g., incubator, centrifuge, lysis equipment) make sample preparation more difficult for personnel with limited skills, few laboratory resources, or in low-resource areas [14].

Since PCR remains to be a gold standard method for detecting NA of viral pathogens, there is a continually increased demand for the development of facile and reliable NA purification and amplification methods to expand infectious disease surveillance activities. Platforms that are rapid, cheap, and applicable in remote settings are of priority and essential for rapid response to limit the spread of pathogens from the point source.

The increase in viral infectious diseases has prompted the use of non-invasive matrices for simplicity of sample collection, cost-effectiveness, and safety. Stool specimen is one of the excellent non-invasive matrices for the detection of various molecular biomarkers, related to infectious and non-infectious diseases of humans and animals [15,16]. It has been used for the detection of influenza A virus (IAV) in birds [17,18], peste des petits ruminants virus in wild and domestic animals [16], porcine epidemic diarrhea virus, and African swine fever virus in pigs [19,20], and astrovirus, rotavirus, norovirus, and hepatitis E virus infections in pigs [21]. However, stool composition makes it one of the most complex and difficult matrices for NA purification and direct amplification, owing to the higher content of PCR inhibitors and debris [22,23]. Bile salts, hemoglobin, polysaccharides, heme, and bilirubin are among those identified to inhibit the amplification process [15,22,23,24,25,26]. The exact mechanism of how some of these inhibitors interfere with the RT process and qPCR is still unrevealed [24,27]. However, it is well known that RNA is easily and frequently degraded by ribonucleases (RNases) from various sources. This proceeds in the process of stool collection from the field to the laboratory, causing the loss of integrity to the viral genetic information for reliable RNA measurements [15]. Therefore, we aimed to capitalize on the hydrogel microparticles’ versatility to develop a simple and user-friendly diagnostic protocol by using microparticles as direct carriers of viral RNA from this complex matrix without prior RNA purification.

This report demonstrates the masterly application of primer-incorporated network (PIN) particles as carriers of viral RNA from stool samples without prior NA purification for qPCR. Acting as discrete reaction compartments, highly porous PINs enable small-sized biomolecules amid complex constituents of the matrix to diffuse into 3D PIN particles’ volume for viral RNA capture and subsequent RT process. Preceded by heat treatment for viral lysis, highly efficient viral RNA capture is accomplished through base pairing hybridization of the viral RNA with the RT primers immobilized onto the crosslinking polymer. The captured viral RNA is readily converted into cDNA, which accumulates and is rightly protected inside the PIN particles. The PIN-cDNA pools can be stored at room temperature, shipped, or directly used to deliver cDNA templates for qPCR. In this work, we have demonstrated high qPCR efficiency detection of the matrix (M) gene for IAV from chicken stool samples using the CFX96 Opus Real-Time PCR System (Bio-Rad Laboratories, Inc. Kaki Bukit, Singapore).

## 2. Materials and Methods

### 2.1. Study Design

We created pseudo-samples to imitate natural stool samples by spiking pure RNA for the M gene or inactivated IAV stock into negative chicken stool samples. Pure RNA was used as our standard to set up the experiment. Where necessary virus stock and stool only were used as positive and no template controls respectively. For the larger part of the study, we used pseudo-samples created by spiking an inactivated H1N1 IAV virus into negative stool samples for method evaluation. Heat-treatment temperature and holding time, matrix dilution, the optimal number of PINs, PIN storage conditions, the efficiency of the assay, and master mix selectivity were the variables of interest. The Ct values were collected and analyzed to evaluate method performance. All amplifications were performed using either ultra-fast real-time PCR (G2-4) System (Micobiomed Co. Ltd., Seoul, Republic of Korea) or CFX96 Opus Real-Time PCR System (Bio-rad Laboratories, Inc. Kaki Bukit, Singapore).

### 2.2. Stool Sample Collection and Preparation

The IAV-negative chicken fecal droppings were collected from a healthy poultry farm and used for the study. Animal handling, non-invasive sample collection procedures, and guidelines for sample collection were adhered to, according to government regulations. Different stool sample concentrations were prepared, 10, 20, 30, and 50% *w*/*v*, to assess the effectiveness of the method against high concentrations of PCR inhibitors and other debris. One gram of stool was dissolved in 10 mL of phosphate buffer saline (PBS) (1 g/10 mL) and used as a standard for this experiment [16,21]. All samples were thoroughly mixed by hand and left for 10 min to sediment at room temperature. The supernatant was aliquoted and kept at –20 °C until the analysis.

### 2.3. Primer and Taqman Probe Design

All oligonucleotides were designed to detect 101 bp of the IAV matrix (M) gene, as previously described [28] (Table 1). The oligos were purified using PAGE purification and purchased from Integrated DNA Technology (IDT), Coralville, IA, USA.

### 2.4. Fabrication of PIN Particles

The pre-polymer solution for both hemisphere and disk type PIN particle fabrication was made by mixing 20% *v*/*v* of UV-photocrosslinkable poly(ethylene glycol) diacrylate (PEGDA) (Sigma-Aldrich, MW700), 40% *v*/*v* of poly(ethylene glycol) (PEG) (Sigma-Aldrich, MW600), 5% *v*/*v* of photo-initiator 2-hydroxy-2-methylpropiophenone (Sigma-Aldrich), and 35% *v*/*v* of PBS (Sigma-Aldrich). The PEG was used to provide porosity of PIN particles to facilitate material transfer during RT and qPCR steps. The 1 mM of the acrydite-forward primer of the IAV M gene was added to the pre-polymer solution to make a final concentration of 50 μM (Table 1). The PIN particles were produced by spotting the pre-polymer solution on pre-patterned polydimethylsiloxane (PDMS) for disk-type PIN particles using a jetting system (Arrayer2000, Advanced Technology Inc., Seoul, Republic of Korea). This was followed by a curing process on ultraviolet (UV) light at 360 nm wavelength, 35 mJ/cm^2^ for 10 sec. The photocrosslinked PIN particles were released from the PDMS surface by mild agitation using PBS buffer containing 0.05% Tween-20 and washed three times with full speed vortex mixer to remove un-crosslinked compounds including acrydite forward primers.

### 2.5. Synthetic RNA Template and Virus Stock Preparation

Pure RNA (1.39 × 10^9^ copies/µL) was synthesized and purchased from IDT, USA (Table 1). Formalin (0.2%) inactivated IAV stock was kindly provided by the avian disease and infectious disease laboratory, at Konkuk University, Seoul, Republic of Korea. The virus with an embryo infectious dose (EID) of 10^8.8^ EID_50_/mL was propagated and titrated in 11-day-old embryonated chicken eggs.

### 2.6. Extraction of Control Viral RNA

Samples were prepared by mixing homogenized stool samples and virus solution in a 1:1 ratio (70 µL stool + 70 µL virus), to make a total volume for extraction of 140 µL. According to the manufacturer instructions the QIAamp Viral RNA Mini Kit (Cat. No 52904) (Qiagen, Hilden, Germany) was used as a standard kit for RNA extraction.

### 2.7. Heat Treatment for Viral Lysis

The suitability of heat treatment for viral lysis and for debris and PCR inhibitors reduction was investigated using spiked RNA and inactivated virus experiments respectively. Stool samples spiked with RNA template (1.39 × 10^9^ copies/µL) or IAV stock was mixed with RiboLock RNase Inhibitor (RI) (Thermal Fisher Scientific Inc. Vilnius, Lithuania) with a ratio of 1:1:0.1 (10 µL virus + 10 µL stool + 1 µL of 2 × RI), for duplicate experiments. Then, the mixture was heat-treated at 100 °C for either 1, 3, 5, 10, 30, or 60 min using a dry heating block (Daihan Scientific Co., Ltd. Seoul, Republic of Korea).

### 2.8. PIN-cDNA Pool Generation and Conventional cDNA Synthesis

The viral RNA was captured through the RT step, utilizing acrydite-modified gene-specific and RT primer immobilized to the PIN particles. A total of 10 PINs were used in each tube and experiments were run in triplicate. The RT reaction mixture contained 2 µL of each 200 U/µL of RevertAid^TM^ RTase (Thermal Fisher Scientific Inc. Vilnius, Lithuania), 1× RI (Thermal Fisher Scientific Inc. Vilnius, Lithuania), dNTPs (Thermal Fisher Scientific Inc. Vilnius, Lithuania), and 5× RT buffer (Thermal Fisher Scientific Inc. Vilnius, Lithuania). The reaction mixture was added into a PCR tube containing PINs particles, and 8 µL of the heat-treated sample was added to make a total reaction volume of 16 µL. For the spin column-based method, the RT reaction mixture contained 2 µL of RTase, 2 µL of dNTPs, 2 µL of RT primer, 2 µL of 5× RT buffer, and 8 µL of RNA. The RT reaction was conducted at 42 °C for 10 min using a dry heating block (Daihan Scientific Co., Ltd. Seoul, Republic of Korea). Different numbers of PIN particles (1, 3, 5, 10, and 50) were used separately, to examine the optimum number of PIN particles required to provide higher assay sensitivity and reliable detection signals.

### 2.9. Conventional and PIN RT-qPCR

All the amplification reactions for solution and PIN particles RT-qPCR were TaqMan probe-based assays. For the heat treatment experiment to alleviate PCR inhibitors, templates were prepared by mixing (1 µL of RNA + 1 µL of stool), then it was diluted to 10%. For the control sample, the stool was replaced with RNase-free water. The reaction mix for solution RT-qPCR contained 1.6 µL of 10 µM for each forward and reverse primer, 1.6 µL of 10 µM probe, 2 µL of template, 1.2 µL distilled water, and 8 µL of one-step 2× RT-PCR Master mix (TaqMan, RNA) (Micobiomed Co. Ltd., Seoul, Republic of Korea). For PIN particles, four particles were inserted into the microfluidic Lab Chip channels, Veri-Q PCR 204 (Micobiomed Co. Ltd., Seoul, Republic of Korea). Then, it was incubated at 4 °C for 30 min with 1.6 µL of 10 µM free reverse primer, 1.6 µL of 10 µM probe, 3 µL of template, 1.8 µL distilled water, and 8 µL of similar PreMix. The PINs were covered with 16 µL of mineral oil to contain fluorescence of amplification signals within individual particles. Thermal cycling conditions were as follows: 42 °C for 10 min, followed by 40 cycles at 95 °C for 8 s, 95 °C for 4 s, and 55 °C for 30 s, with combined annealing and extension steps. All amplifications were performed using Ultra-fast real-time PCR (G2-4) System (Micobiomed Co. Ltd., Seoul, Republic of Korea). For the spin column-based qPCR using CFX96 Opus Real-Time PCR System (Bio-Rad Laboratories, Inc. Kaki Bukit, Singapore). The reaction mix for qPCR contained 1.6 µL of 10 µM for each forward and reverse primer, 1.6 µL of 10 µM probe, 3.2 µL of template, and 8 µL of the TaqMan^TM^ Universal PCR Master Mix II (Applied Biosystems, Vilnius, Lithuania). Thermal cycling conditions were as follows: initial denaturation at 95 °C for 10 min, followed by 40 cycles at 95 °C for 15 s, and 60 °C for 1 min, with combined annealing and extension steps.

### 2.10. Viral RNA Delivery for qPCR

The PIN particles with a cDNA pool were briefly rinsed using PBS with 0.05% Twin 20 and transferred into a new PCR tube. The qPCR reaction mixture contained 1.6 µL of each 10 µM of TaqMan probe, forward, and reverse primer, 3.2 µL of RNase-free water, and 8 µL of the TaqMan^TM^ Universal PCR Master Mix II (Applied Biosystems, Vilnius, Lithuania), was added to a PCR tube containing PIN-cDNA pools. The presence of a free forward primer allows the qPCR reaction to take place in an aqueous medium, utilizing the cDNA template released from the PINs into the reaction mixture during the denaturation step of the qPCR. Thermal cycling conditions were as follows: initial denaturation at 95 °C for 10 min, followed by 40 cycles at 95 °C for 15 s, and 60 °C for 1 min, with combined annealing and extension steps using the CFX96 Opus Real-Time PCR System (Bio-Rad Laboratories, Inc. Kaki Bukit, Singapore).

### 2.11. Limit of Detection (LoD) and PCR Efficiency

A parallel examination was conducted by comparing the sensitivity and LoD between PIN-based assay and conventional qPCR following viral RNA extraction using QIAamp Viral Mini Kit. The virus stock solution was 10-fold serially diluted from 10^0^ to 10^6^, then mixed at a 1:1 ratio with homogenized negative stool samples for the experiment. Four replicates of similar experiments were conducted with duplicate samples for each method. Virus stock and stool only were used as positive and no-template controls (NTC), respectively.

### 2.12. PIN-cDNA Pool Storage Conditions

Two sets of PIN-cDNA pools were stored at room temperature and 4 °C for 2 months (*n* = 36). Each set had one subset of rinsed PIN-cDNA pool with PBS containing 0.05% Twin 20 to remove remnants of stool sample and other RT reagents. While, the other subset was left without rinsing, to examine the effect of stool residues and other RT reagents on the stability of PIN-cDNA pools and to deduce their proper storage condition and duration. One PIN-cDNA pool from each subset was used for qPCR on days 1, 2, 3, 5, 10, 15, 30, 45, and 60, respectively. The Ct values were monitored to assess the degradation of carried cDNA in each set of PIN-cDNA pools. Gel electrophoresis was run to confirm the presence of the expected band size.

### 2.13. Gel Electrophoresis

Agarose gel electrophoresis was conducted using 3% of NuSieve^TM^ 3:1 Agarose (Lonza, Rockland, USA) with 1×TBE buffer (Bioneer Corp, Daejeon, Republic of Korea). The gel was stained with 3 µL of SYBR^®^ Safe DNA gel stain (Invitrogen, Carlsbad, CA, USA). About 5 µL of qPCR amplicons were loaded into the gel, and the DNA bands were observed using Safe Imager^TM^ 2.0 blue-light transilluminator against Ultra Low Range DNA (100 bp) Ladder (Invitrogen, Carlsbad, CA, USA). 

### 2.14. Nucleotide Sequencing

A total of 4 DNA bands (101 bp) for the M gene of the H1N1 strain obtained, were sent to Bioneer Corporation (Daejeon, Republic of Korea) for nucleotide genome sequencing. The chromatograms for both forward and reverse primers were read and assessed for their quality using Sequence Scanner software v2.0 (Applied Biosystems, Foster, CA, USA), and the consensus sequences were assembled using BioEdit software v7.2. The nucleotide identity of the viral sequences was verified using BLASTn search against the NCBI GenBank database.

### 2.15. Data Analysis

All the raw data were automatically extracted in Microsoft Office-Excel 2016 (Microsoft, Seattle, WA, USA), from the Ultra-fast real-time PCR (G2-4) System (Micobiomed Co. Ltd., Seoul, Republic of Korea) and the CFX96 Opus Real-Time PCR System (Bio-rad Laboratories, Inc. Kaki Bukit, Singapore) computer software. We applied simple descriptive statistics (Averages) to analyze our data. We also applied linear regression to find a correlation between Ct values and template concentration for both methods. The originLab^®^2020 software, Northampton, MA, USA), and Epi info^TM^ version 7.2.4.0 software (CDC, Atlanta, GA, USA) were used for data analysis and drawing the curves.

## 3. Results and Discussion

### 3.1. Principle of the Assay

In this study, stool samples are analyzed through a seven-step protocol (Figure S1). First, the viral RNA is purified from the matrix using validated spin-column RNA purification kits. Then, the eluted viral RNA is used to synthesize cDNA at a uniform temperature. Lastly, the resulting cDNA can be stored preferably at −20 °C for future use, or directly utilized as a template for the subsequent qPCR in a separate reaction tube.

**Figure 1:**
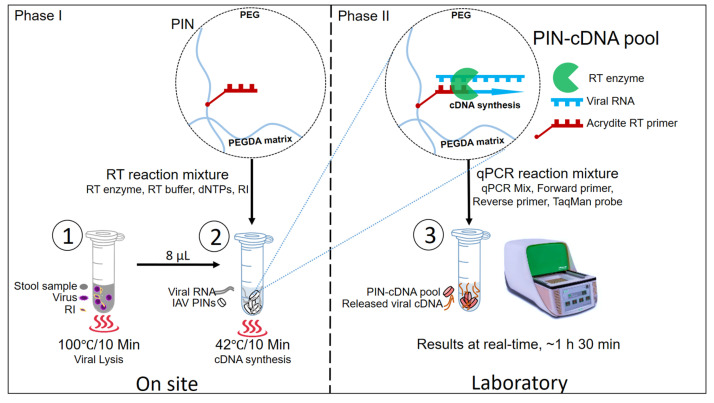
The schematic presentation of three main steps for PIN particles as viral RNA carriers for qPCR.

In the proposed novel protocol, there are two main phases with three hands-on steps. The three main hands-on steps are viral lysis, PIN-cDNA pool synthesis, and qPCR (Figure 1). Phase one: the field phase involves viral lysis and viral PIN-cDNA pool synthesis. This can easily be conducted on-site using a compact heater, a newly designed 12 V portable thermostat device with dual plates for viral lysis and PIN-cDNA pool synthesis. Phase two is the laboratory phase, with only one hands-on activity in the laboratory, where the PIN-cDNA pools are transferred to PCR tubes for subsequent qPCR. Step 1: Heat treatment is applied to homogenized stool samples for viral lysis at 100 °C for 10 min. This is an important step that releases the viral RNA out of the nucleocapsid protein of IAV. Step 2: A heat-treated stool sample is mixed with 10 PIN particles and an RT reaction mixture for the RT process at 42 °C for 10 min. The first-strand cDNA is synthesized by priming with immobilized RT primers which are gene-specific using RTase, an RNA-directed DNA polymerase. The whole process results in highly enriched PIN-cDNA pool formation, which can either be stored for long-term use or directly used to deliver the cDNA templates for qPCR. Step 3: The PIN-cDNA pools as a rich source of viral cDNA templates are loaded with a qPCR reaction mixture for the qPCR step. During the denaturation step, where the temperature rises to 95 °C, it causes the attached double-stranded cDNA to melt, and single-stranded DNA templates are released for aqueous-phase qPCR. In general, the current method has significantly reduced the hands-on steps by cutting down the number of sample processing steps to three (Figure S1), and there is no need of using a centrifuge. Thus, escaping the viral RNA purification process makes this method more convenient for diagnostic and research purposes.

### 3.2. Structural Characteristics of PIN

**Figure 2:**
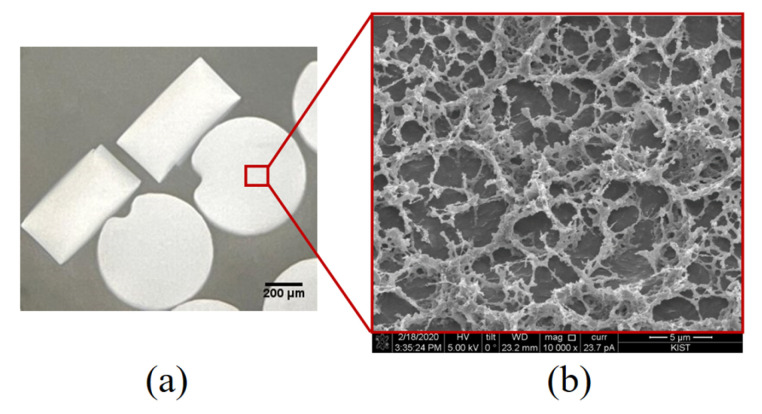
Structural characteristics of PIN; (**a**) A pictogram of a swollen disk-shaped PIN particle suspended in PBST buffer captured using an inverted microscope, 20× magnification; (**b**) Cryo-SEM (10,000×) showing the internal structure of the PIN particle.

We fabricated disk-shaped PIN particles with fixed sizes to facilitate stable handling procedures. The PIN particles had an average diameter of approximately 630 µm, ranging from 626 µm to 634 µm. Their height ranged from 318 µm to 334 µm, with an average height of 328 µm (Figure 2a). The approximate volume of a single disk PIN is about 0.1 µL. The inner PIN morphology was studied using scanning electron microscopy (SEM). The SEM revealed highly spacious inner structures made by PEGDA, which allows the flow of aqueous media across the PIN and the availability of a large surface area for efficient RNA capture through hybridization and PIN-cDNA pool formation (Figure 2b). It is assumed that the porous structure reduces the entry of small particulates into the PIN volume, keeping the enzymatic reaction stable for efficient viral RNA capture amid other stool components. Each PIN with an abundant concentration of covalently immobilized RT primers will capture a sufficient amount of viral RNA without NA purification. Utilizing these PIN microparticles as a filter-integrated RT reservoir and carriers of viral cDNA, the three steps of this novel protocol were optimized.

### 3.3. Heat Treatment for Viral Lysis: The First Step

The most widely used method is chemical lysis; however, for a simpler protocol, we chose to use thermal treatment. It is well-known that heat energy denatures the membrane proteins and lyses the cells to release intracellular material, such as RNA. In addition, adverse enzyme reactions are subsided, while proteins and other biomolecules are massively deformed [29,30]. The aliquots of stock virus solution were heat-treated at 60 °C, 80 °C, 100 °C, and 120 °C for 1 min, 3 min, 5 min, and 10 min to find the optimal temperature and holding time for viral lysis. The heat-treated stock virus samples were directly used as a template for PIN particle-based RT-qPCR and the Ct values were assessed. We found that heat treatment was effective for viral lysis and ensured viral RNA is released from the viral nucleocapsid protein ready for the RNA capture and RT process. The optimum temperature at 100 °C for 3 min was sufficient to provide high amplification signal intensity and stable Ct values. The introduction of heat intervention improved the viral lysis effect by approximately 2 logs. The Ct values were pulled from 27.79 of the control sample to 22.39 following the effect of heat treatment for viral lysis (Table 2). The lower temperatures below 100 °C displayed worse sensitivity despite a time increase from 1 to 10 min, while the higher temperature had no additional effect. Despite showing the best results by an average of 1 Ct units, 120 °C was avoided due to difficulties in sample handling and maintenance of stable heating temperature, and for increased safety risks. These findings are supported by several other studies; where human stool samples were boiled for 15 min to achieve viral lysis for microparticles-based RT-qPCR [31,32]. Similarly, it was proved possible to escape NA purification by applying heat treatment on oropharyngeal swab samples (98 °C for 5 min) and nasopharyngeal swabs (95 °C for 10 min) in combination with 10% dilution of the matrix for detection of severe acute respiratory syndrome coronavirus 2 [33,34].

**Figure 3:**
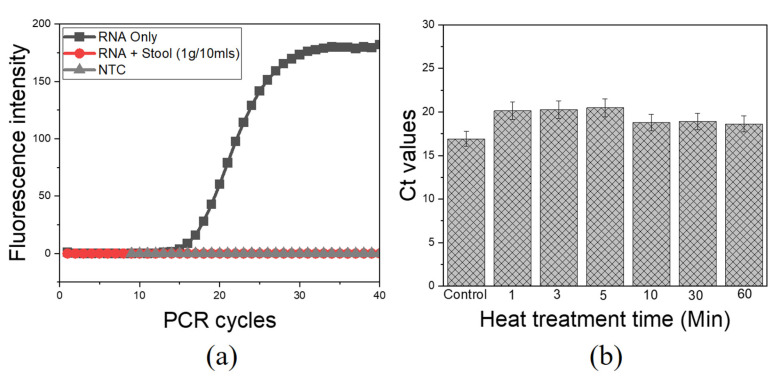
PCR inhibitors and the effect of heat treatment for direct RNA detection from stool samples. An aqueous phase qPCR using ultra-fast real-time PCR (G2-4) System; (**a**) Complete PCR inhibition of pure RNA spiked into stool sample without heat treatment; (**b**) Heat treatment is effective in suppressing heat-sensitive PCR inhibitors/debris. NTC, RNase-free water. Control, pure RNA.

The major concern with direct viral RNA capture and the following RT process is the presence of various PCR inhibitors in stool samples as earlier mentioned. There was clear evidence for complete inhibition of aqueous-phase one-step RT-qPCR when pure RNA was spiked into stool samples without heat intervention, as compared to pure RNA as control (Figure 3a). Likewise, qPCR was completely inhibited when pure DNA was spiked into a similar matrix (Figure S2). These findings confirmed the presence of active PCR inhibitors in this matrix, with a drastic inhibitory effect on the amplification process as previously reported [15,27]. The introduction of heat treatment adopted for viral lysis had profound results in suppressing the effect of heat-labile PCR inhibitors. The heat treatment at 100 °C for 10 min was sufficient to overcome the inhibition effects to obtain clear amplification signals with an average Ct of 18.78, as compared with pure RNA as control with an average Ct of 16.9 (Figure 3b). Following these results, the extended heat treatment condition (100 °C for 10 min) was selected, based on its effectiveness for both viral lysis and deactivation of heat-labile PCR inhibitors.

In addition, it was necessary to consider the NA degradation threat posed by both exogenous and endogenous RNases, which can contaminate samples during collection, shipping, and analysis, or are naturally found within the matrix [15]. The use of RI was considered essential to protect viral RNA from RNases during sample processing and to ensure there is an adequate copy number of intact viral RNA amid other stool components available for analysis [32]. These guaranteed viral RNA measurements are more reliable and proceed at desirable efficiency.

### 3.4. Viral RNA Capture and cDNA Synthesis: The Second Step

To increase the RNA analysis success rate, a sufficient amount of viral RNA must be captured by PIN particles. Stool samples were suspended in an aqueous buffer at the concentration of 1 g/10 mL (10%, *w*/*v*) as standard [16,21,25]. However, we also prepared thicker suspensions of 2 g/10 mL, 3 g/10 mL, and 5 g/10 mL to simulate harsh conditions from heavy contaminations.

**Figure 4:**
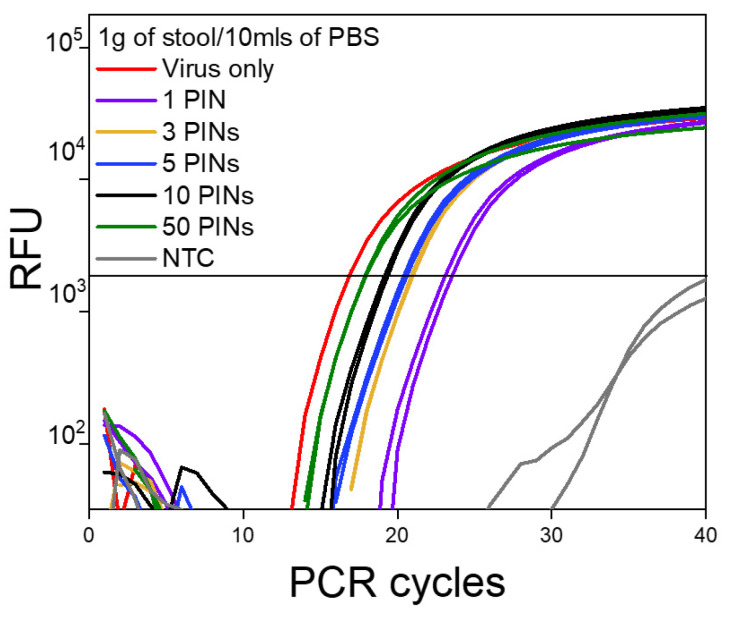
The log amplification curve for 1 g/10 mL of stool samples against the number of PIN particles used (*n* = 15).

Starting with 1 g/10 mL as a standard dilution for this matrix (Figure 4), and up to 5 g/10 mL of PBS buffer (Figure S3), we were able to obtain clear amplification signals using PIN-cDNA pool assay. The combination of heat treatment and RI application enabled efficient enzymatic reaction for viral RNA capture without prior RNA purification. The assay sensitivity is preferable with a more dilute matrix (1 g/10 mL) which consistently has displayed lower Ct values by an average of 1.1 Ct units, compared to thicker stool suspensions (5 g/10 mL). The Ct difference observed between the two sets of samples was relatively not significant (Table 3). This means viral RNA capture can be performed without precise weight measurements of the matrices for reliable analyses of the biomarkers for up to 5 g/10 mL. The ability to capture viral RNA using synthesized PIN-cDNA pools in thicker and non-centrifuged stool suspensions would be advantageous for on-site surveillance activities using non-invasive and complex matrices. In addition, the slight Ct difference between the diluted and thicker stool suspensions might be a result of the existence of significant levels of PCR inhibitors in the thicker stool suspensions [15,22,27].

Regardless of the matrix concentration, a single PIN particle was sufficient to produce an enriched PIN-cDNA pool for efficient qPCR performance. In general, there was a significant enhancement in the detection of viral RNA when the number of PIN particles was increased from 1 to 10. The Ct values dropped substantially by an average of 4 Ct units (>1 log), from 23.79 to 19.84 for 1 and 10 PIN particles, respectively (Table 3). A further increase in the number of PIN particles had less impact on the assay sensitivity, as the Ct was dropped by an average of 1.6 Ct units (< 1 log); from 19.84 for 10 PINs to 18.24 for 50 PIN particles. The aforementioned observation provides method and sensitivity versatility as the number of PIN particles can be adjusted based on the assay requirements. In this case, 10 PIN particles were most likely sufficient for viral RNA capture and delivery for an efficient and sensitive qPCR.

**Figure 5:**
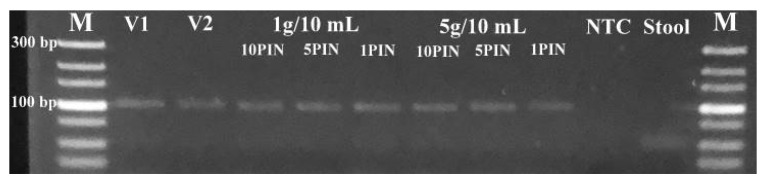
Agarose gel electrophoresis pictogram showing the correct DNA band sizes for both low and high stool concentration. Lane M, Ultra-low range DNA (100 bp) ladder; Lane 1–2, the virus only; Lane 3–8, virus + stool; Lane 9, RNase-free water; Lane 10, stool sample only.

The amplicons were confirmed by examining the DNA band sizes after qPCR using gel electrophoresis. All the experiments generated the expected band size (101 bp), except for stool only and NTC which had no DNA bands (Figure 5). Generation of the correct band size with quality to support DNA sequencing means that the assay can provide quite reliable test results. Following genome sequencing and NCBI BLASTn search to confirm the amplicon specificity, all four sequenced samples were identified as the M gene of the swine H1N1 IAV strain (MN700055.1). The PIN-cDNA pools developed in this study could be useful for next-generation sequencing and molecular epidemiological studies of clinical samples.

### 3.5. The qPCR and Limit of Detection: The Third Step

**Figure 6:**
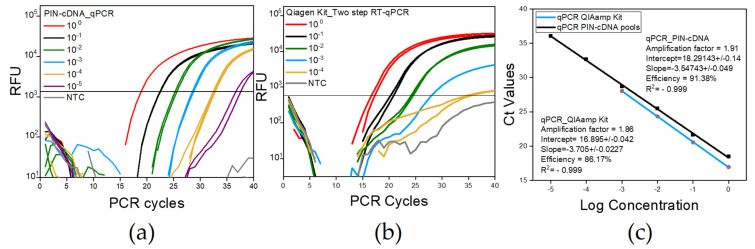
The LoD and PCR efficiency differences between the two methods; figures show one representative experiment of the four replicates conducted each with duplicate samples. (**a**) A curve for qPCR using PIN-cDNA pool; (**b**) A curve for qPCR using spin column-based Kit; (**c**) The standard curve showing the Ct values for qPCR with serial dilution having a constant interval of 3.5 and 3.7 for the Ct value for PIN-cDNA pool and column-based kit respectively. The dilution 10^−6^ and 10^−5^ for Figure 6a,b, respectively, yielded a negative result in the first experiment, and, therefore, was not included in the following experiments.

The LoD and qPCR efficiency were examined to evaluate the qPCR protocol developed in this study. The qPCR using PIN-cDNA pools was conducted in comparison with a total RNA extraction kit which was followed by two-step RT-qPCR. The LoD was as low as 2 × 10^−5^ of the stock virus dilution when PIN-cDNA pools were used together with TaqMan^TM^ Universal PCR Master Mix II (Applied Biosystems, Vilnius, Lithuania) (Figure 6a). It was lower than that of the spin column-based assay with 2 × 10^−4^ (Figure 6b). The results followed the use of a 10-fold serially diluted virus stock solution (10^8.8^ EID_50_/mL) mixed with negative stool samples in a ratio of 1:1, with the same composition of the RT mixture for both assays. The use of PIN-cDNA pools for qPCR also displayed a better qPCR efficiency of about 91.38%, as compared to the spin column-based assay with a qPCR efficiency of about 86.17% (Figure 6c). In addition, signals from viral RNA samples were more clearly distinguished from the ones from NTC using PIN-cDNA pools for qPCR. We assume that the delivery of cDNA, separated from the components of the RT reaction, secured the stable enzyme reaction for qPCR. The conventional method of the spin column was observed to concentrate NA by 2 folds, but the analytical performance of the novel method is either equivalent or rather improved (Table 4). These findings suggest that RT reaction within the PIN is effectively conducted even in a harsh environment containing components of the stool matrix.

Despite the observed Ct value differences, the use of PIN-cDNA pools for qPCR has shown a high tendency for selectivity and consistency in results even with low target concentration samples. Having a comparable LoD range with a much simpler protocol implies that the use of PIN-cDNA pools is more advantageous for diagnostic and research purposes than conventional methods.

### 3.6. Storage Conditions for PIN-cDNA Pools

**Figure 7:**
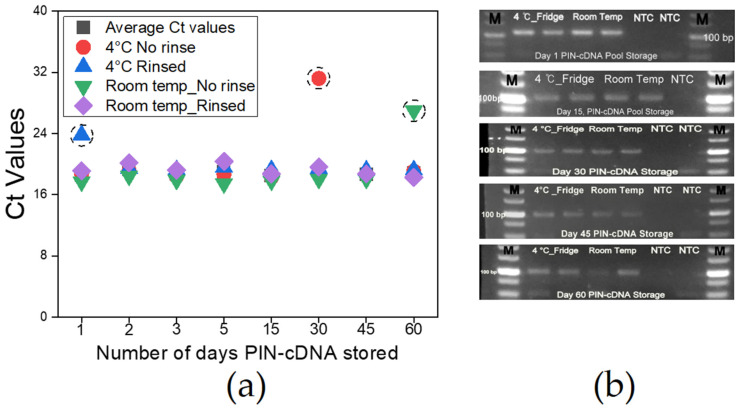
PIN-cDNA pools storage conditions and duration (*n* = 36); (**a**) A curve showing Ct values for different PIN-cDNA pools stored at four different conditions; (**b**) Agarose gel electrophoresis showing expected DNA band sizes after storage for specific time intervals.

Proper storage of matrices before RNA extraction and storage of RNA thereafter requires a highly stable cold chain system to maintain the molecules at a stable state, which is instrumental for reliable qPCR results [7]. Likewise, the need for accurate and intact viral genetic information carried by the PIN-cDNA pool was necessary to be monitored, especially for longer storage duration to assure the reliability of test results over time. We stored PIN-cDNA pools (*n* = 36) under different conditions for 2 months while assessing at intervals the quality and quantity of the cDNA pool by qPCR and gel electrophoresis. We find that PIN-cDNA pools can be stored either at room temperature or 4 °C for 2 months while soaked in PBS containing 0.05% Tween 20 without cDNA deterioration. Furthermore, we noted that rinsing the PIN-cDNA pools after the RT step to remove the remnants of stool, and other RT reaction elements do not affect the quality of cDNA in the PIN particles. Similar observations were made when the PIN-cDNA pools were not rinsed. The two sets of PIN-cDNA pools maintained a stable pattern of Ct values and amplification signal intensity for the whole period of storage, for over 90% of the cases (Figure 7a). This is advantageous for downstream applications including qPCR and sequencing. This is ample time that paves the way for an extensive onsite viral RNA capture, using a 12 V portable heat-treatment device. The prototype device with two heating plates provides room for concurrent heat treatment for viral lysis and RT process (Figure S4). The extensive PIN-cDNA stability at room temperature is an important feature that supports this technology for onsite viral RNA capture, to improve molecular epidemiology studies and surveillance activities for zoonotic infectious diseases using non-invasive matrices.

## 4. Conclusions

As proof of concept, the PIN particles showed a high ability to capture viral RNA from stool samples without the use of a centrifuge. In addition, the assay has displayed high qPCR efficiency, good linearity, and comparable LoD with a commercialized viral RNA purification kit. The storage duration of the PIN-cDNA pool at room temperature is another powerful characteristic of the PIN particle technology for onsite applications to support disease surveillance activities using non-invasive and complex matrices. However, it has been difficult to validate the method with field clinical samples, especially assay specificity using known negative samples or positive samples for other viruses. We are optimistic that the use of pseudo-samples has managed to depict its diagnostic potential. The method will also benefit from further optimization and validation procedures, and some minor modifications (e.g., lyophilized RT reagents and PIN pre-loaded tubes), which will facilitate easy onsite application.

## Figures and Tables

**Table 1:** Oligonucleotide primers, probes, and templates used in this study.

Species	Type	Sequence Information (5′–3′)	Size
IAV H1N1, M gene	F:	Acrydite-AGATGAGTCTTCTAACCGAGGTCG	101 bp
	R:	TGCAAAAACATCTTCAAGTCTCTG	
	P:	6-FAM-TCAGGCCCCCTCAAAGCCGA-BHQ_1	
	T:	TGCAAACACATCTTCAAGTCTCTGCGCGATCTCGGCTTTGCGGGGGCCTGACGGGACGATAGAGAGAACGTACGTTTCGTCCTCGGTTAGAAGACTCATCT	

Legend: F is a forward primer, R is a reverse primer, and P is a single quenched TaqMan probe consisting of 6-FAM molecule as reporter dye at 5′ end, and BHQ-1 as a quencher at the 3′ end of the oligonucleotide. T is amplicon information of the targets.

**Table 2:** Heat treatment for viral lysis.

		Average Ct Values							
Temperature		60 °C	SD	80 °C	SD	100 °C	SD	120 °C	SD
Time in minutes	1	30.11	1.97	28.75	2.01	25.54	0.62	22.74	0.24
	3	27.83	0.56	25.36	0.07	22.39	0.72	21.50	0.87
	5	30.67	2.33	25.18	0.40	22.62	0.82	21.46	0.70
	10	27.76	1.26	24.69	0.67	22.23	0.16	-	-
Control; No heat treatment Ct = 27.79 SD, 0.50									

**Table 3:** The number of PIN particles against matrix concentration.

	Average Ct Values			
Number of PIN Particles	1 g of Stool/10 mL	SD	5 g of Stool/10 mL	SD
1 PIN	23.25	0.20	24.32	0.33
3 PINs	20.90	0.06	22.22	0.20
5 PINs	20.41	0.09	21.53	0.43
10 PINs	19.20	0.09	20.47	0.30
50 PINs	17.88	0.04	18.60	0.35

**Table 4:** The Ct values for a standard curve from a representative experiment.

	Dilution	10^0^	10^−1^	10^−2^	10^−3^	10^−4^	10^−5^
Ct values	PIN-cDNA pool	18.48	21.64	25.44	28.69	32.66	36.05
	QIAamp	16.93	20.54	24.32	28.02	35.64	-

## Data Availability

The data presented in this study are available on request from the corresponding author. The data are not publicly available due to privacy.

## Supplementary Materials

The following supporting information can be downloaded at: https://www.mdpi.com/article/10.3390/diagnostics13020261/s1, Figure S1: Schematic presentation of the differences between the two protocols. The PIN-cDNA pool qPCR protocol (top), and Spin-column RNA preparation (bottom); Figure S2: Complete qPCR inhibition of pure DNA spiked in a stool sample (1 g/10 mL) without any pre-treatment procedure. The aqueous phase qPCR was conducted in duplicate using ultra-fast real-time PCR (G2-4) System; Figure S3: The log amplification curve for 5g/10 mL of stool samples against the number of PIN particles used (*n* = 15); Figure S4: A prototype heating device with 10 sample wells in each plate. Plate 1 for heat treatment at 100 °C; Plate 2 for RT step at 42 °C.
