# Supplementary material for: Microparticles as Viral RNA Carriers from Stool for Stable and Sensitive Surveillance
Source: Diagnostics (Basel). 2023 Jan 10;13(2):261. doi: 10.3390/diagnostics13020261 (PMC9857651; doi:10.3390/diagnostics13020261)
Supplement: Supplementary file 1 [file diagnostics-13-00261-s001.zip › diagnostics-1985685-supplementary.pdf]

Supporting Information

Microparticles as Viral RNA Carriers from Stool for Stable and Sensitive Surveillance

Emmanuel George Kifaro[1,2,3], Mi Jung Kim[1], Seungwon Jung[1], Yoon-ha Jang[1], Sungyeon Moon[1], Dong-Hun Lee[4], Chang-Seon Song[4], Gerald Misinzo[2,3] and Sang Kyung Kim[1,5*]

[1] Molecular Recognition Research Center, Korea Institute of Science and Technology (KIST), 5, Hwarang-ro 14-gil, Seongbuk-gu, Seoul 02792, Republic of Korea

[2] Department of Veterinary Microbiology, Parasitology, and Biotechnology, Sokoine University of Agriculture (SUA), PO Box 3019, Morogoro, Tanzania

[3] Southern African Centre for Infectious Disease Surveillance (SACIDS), Africa Centre of Excellence for Infectious Diseases of Humans and Animals in Eastern and Southern Africa (ACE), Sokoine University of Agriculture (SUA), PO Box 3297, Morogoro, Tanzania

[4] College of Veterinary Medicine, Konkuk University, 120 Neungdong-ro, Gwangjin-gu, Seoul 05029, Republic of Korea

[5] KHU-KIST Department of Converging Science and Technology, Kyung Hee University, Seoul 02447, Republic of Korea

***Corresponding author:** Sang Kyung Kim, Ph.D.

Email:
sangk@kist.re.kr
0000-0002-1186-8302

The Difference Between the Two Protocols

The current method has significantly reduced the hands-on steps by cutting down the number of sample processing steps to three, which are namely; a) viral lysis b) PIN-cDNA pool synthesis, and c) qPCR. Compared to the spin-mini methods, RNA preparation alone has about six steps which include several tube changes, vortex mixing, and centrifuging steps. In general, both methods require a minimum of 30 min to accomplish either PIN-cDNA pool generation or RNA purification steps.

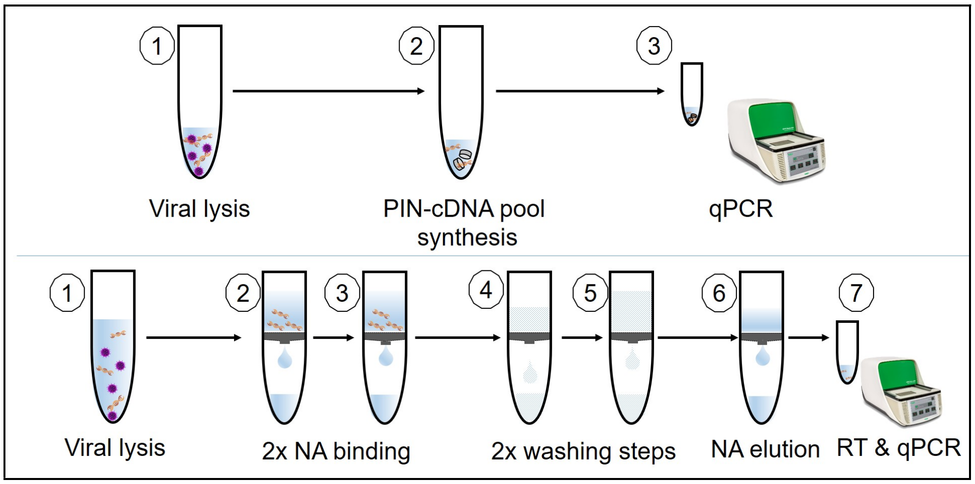

Figure S1. Schematic presentation of the differences between the two protocols. The PIN-cDNA pool qPCR protocol (top), and Spin-column RNA preparation (bottom).

The Stool Samples Contain PCR Inhibitors

One-step RT-qPCR was completely inhibited when synthetic RNA spiked with negative stool samples (1g/10 mL) was analyzed without heat intervention (n=4). Similarly, qPCR was inhibited as synthetic DNA was spiked into a similar matrix. The findings confirmed the presence of active PCR inhibitors in this matrix, with a drastic inhibitory effect on the amplification process as previously reported [1, 2]. The introduction of heat treatment adopted for viral lysis had profound results in suppressing the effect of heat-labile PCR inhibitors. Heat treatment at 100 °C for 10 min was sufficient to overcome the inhibition

effects to obtain clear amplification signals. The DNA sample was 100-fold diluted (red curve) in reference to DNA only as control (black curve) without dilution.

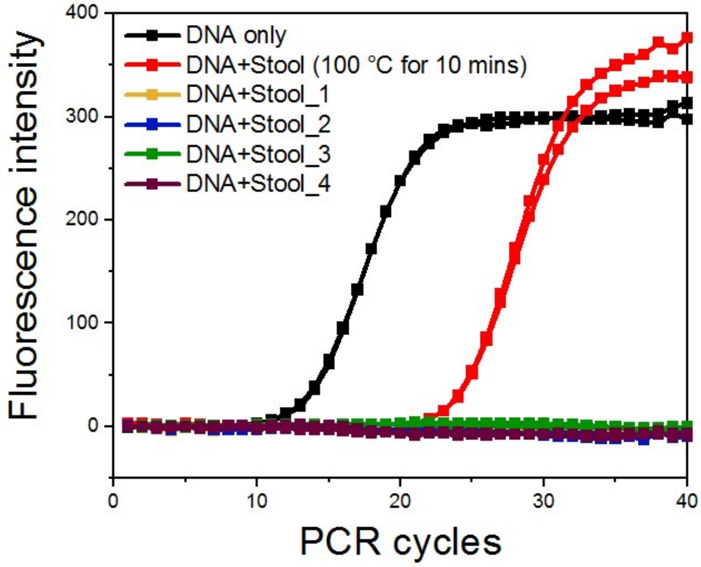

Figure S2. Complete qPCR inhibition of pure DNA spiked in a stool sample (1g/10 mL) without any pre-treatment procedure. The aqueous phase qPCR was conducted in duplicate using ultra-fast real-time PCR (G2-4) System.

Viral RNA Detection Against High Stool Concentration

Stool samples are generally suspended in an aqueous buffer at the concentration of 1g/10 mL (10%, w/v) as standard. We were able to obtain clear amplification signals using PIN-cDNA pool assay with thicker stool suspension of 5g/10 mL of PBS buffer. Although the assay sensitivity was preferable with 1g/10 mL stool suspension, the ability to capture viral RNA and synthesize PIN-cDNA pools against thicker and non-centrifuged stool suspensions is an added advantage to the current method.

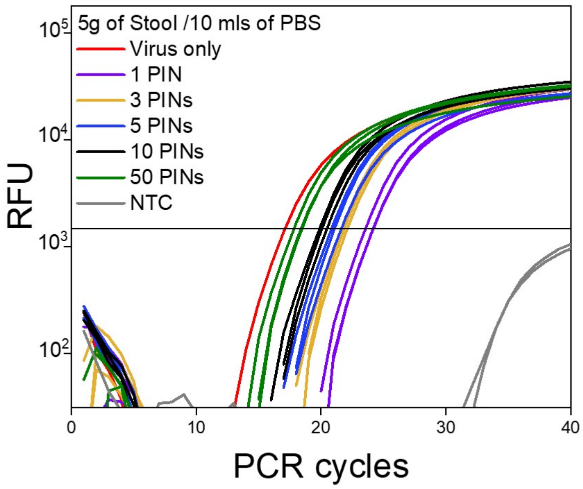

Figure S3. The log amplification curve for 5g/10 mL of stool samples against the number of PIN particles used (n=15).

The Prototype Heating Device

The compact heater, a newly designed 12V portable thermostat device with dual plates, provides room for concurrent heat treatment for viral lysis and PIN-cDNA pool synthesis. The device was used in parallel with validated commercially available dry heating blocks.

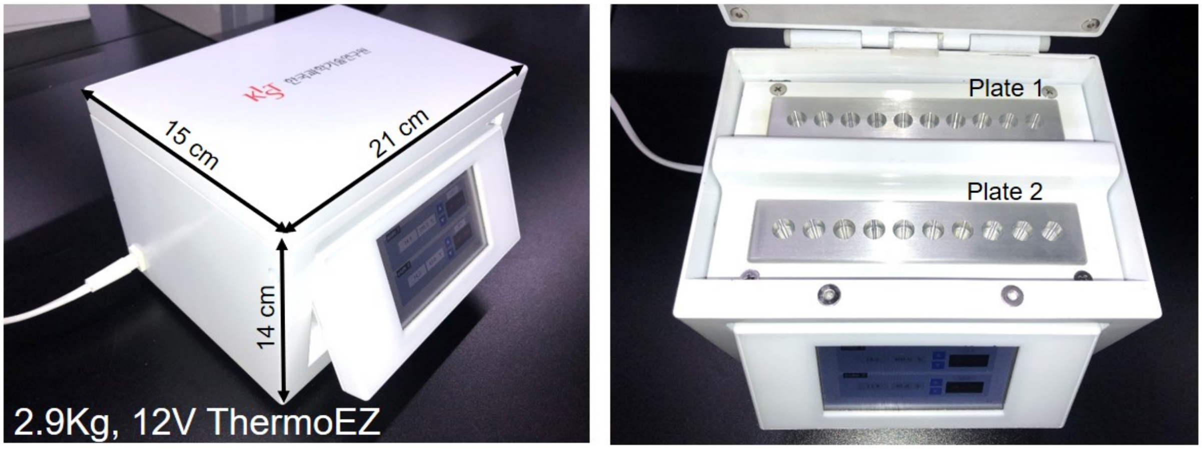

Figure S4. A prototype heating device with 10 sample wells in each plate. Plate 1 for heat treatment at 100 °C; Plate 2 for RT step at 42 °C.

References

1. Das, A.; Spackman, E.; Pantin-Jackwood, M. J.; Suarez, D. L., Removal of real-time reverse transcription polymerase chain reaction (RT-PCR) inhibitors associated with cloacal swab samples and tissues for improved diagnosis of Avian influenza virus by RT-PCR. *Journal of Veterinary Diagnostic Investigation* **2009,** *21* (6), 771-778.

2. Nechvatal, J. M.; Ram, J. L.; Basson, M. D.; Namprachan, P.; Niec, S. R.; Badsha, K. Z.; Matherly, L. H.; Majumdar, A. P.; Kato, I., Fecal collection, ambient preservation, and DNA extraction for PCR amplification of bacterial and human markers from human feces. *J Microbiol Methods* **2008,** *72* (2), 124-32.
